# Relaxin deficiency results in increased expression of angiogenesis- and remodelling-related genes in the uterus of early pregnant mice but does not affect endometrial angiogenesis prior to implantation

**DOI:** 10.1186/s12958-016-0148-y

**Published:** 2016-03-22

**Authors:** Sarah A. Marshall, Leelee Ng, Elaine N. Unemori, Jane E. Girling, Laura J. Parry

**Affiliations:** School of BioSciences, The University of Melbourne, Royal Parade, Parkville, Victoria Australia; Corthera Inc., San Mateo, California USA; Gynaecology Research Centre, Department of Obstetrics and Gynecology, The University of Melbourne and Royal Women’s Hospital, Parkville, Victoria Australia

**Keywords:** Relaxin, Angiogenesis, Early pregnancy, Uterus, Progesterone

## Abstract

**Background:**

Extensive uterine adaptations, including angiogenesis, occur prior to implantation in early pregnancy and are potentially regulated by the peptide hormone relaxin. This was investigated in two studies. First, we took a microarray approach using human endometrial stromal (HES) cells treated with relaxin *in vitro* to screen for target genes. Then we aimed to investigate whether or not relaxin deficiency in mice affected uterine expression of representative genes associated with angiogenesis and uterine remodeling, and also blood vessel proliferation in the pre-implantation mouse endometrium.

**Methods:**

Normal HES cells were isolated and treated with recombinant human relaxin (10 ng/ml) for 24 h before microarray analysis. Reverse transcriptase PCR was used to analyze gene expression of relaxin and its receptor (*Rxfp1*) in ovaries and uteri; quantitative PCR was used to analyze steroid receptor, angiogenesis and extracellular matrix remodeling genes in the uteri of wild type (*Rln+/+*) and *Rln-/-* mice on days 1-4 of pregnancy. Immunohistochemistry localized endometrial endothelial cell proliferation and mass spectrometry measured steroid hormones in the plasma.

**Results:**

Microarray analysis identified 63 well-characterized genes that were differentially regulated in HES cells after relaxin treatment. Expression of some of these genes was increased in the uterus of *Rln+/+* mice by day 4 of pregnancy. There was significantly higher vascular endothelial growth factor A (*VegfA*), estrogen receptor 1 (*Esr1*), progesterone receptor (*Pgr*), *Rxfp1*, egl-9 family hypoxia-inducible factor 1 (*Egln1*), hypoxia inducible factor 1 alpha (*Hif1α*)*,* matrix metalloproteinase 14 (*Mmp14*) and ankryn repeat domain 37 (*Ankrd37*) in *Rln*-/- compared to *Rln+/+* mice on day 1. Progesterone receptor expression and plasma progesterone levels were higher in *Rln-/-* mice compared to *Rln+/+* mice. However, endometrial angiogenesis was not advanced as pre-implantation endothelial cell proliferation did not differ between genotypes.

**Conclusions:**

Relaxin treatment modulates expression of a variety of angiogenesis-related genes in HES cells. However, despite accelerated uterine gene expression of steroid receptor, progesterone and angiogenesis and extracellular matrix remodeling genes in *Rln-/-* mice, there was no impact on angiogenesis. We conclude that although relaxin deficiency results in phenotypic changes in the pre-implantation uterus, endogenous relaxin does not play a major role in pre-implantation angiogenesis in the mouse uterus.

**Electronic supplementary material:**

The online version of this article (doi:10.1186/s12958-016-0148-y) contains supplementary material, which is available to authorized users.

## Background

To start a pregnancy, the endometrium must be in a receptive state for embryo implantation. In women, the endometrium undergoes extensive vascular growth, remodeling, and regression on an approximately monthly basis [1]. During the proliferative phase of the cycle, the endometrium and its associated vasculature undergo a period of rapid growth, with subsequent maturation occurring in the secretory phase [2]. Failure of the endometrium to appropriately remodel and vascularize can result in miscarriage and other pregnancy complications such as fetal growth restriction [3, 4].

Growth, maturation and remodeling of the endometrium and its blood vessels are primarily regulated by the ovarian steroid hormones estrogen and progesterone [5, 6]. In mice, endometrial endothelial cell proliferation increases prior to embryo implantation in response to increasing plasma progesterone concentrations. Progesterone treatment in ovariectomized mice stimulates angiogenesis; estrogen priming moderates this progesterone-induced endothelial cell proliferation [6]. The peptide hormone relaxin also contributes to remodeling in early pregnancy in primates [7]. Although primarily ovarian in origin, relaxin is also produced by other tissues during pregnancy, including the endometrium, where it is hypothesized to exert paracrine and autocrine uterotrophic effects [8, 9]. Relaxin treatment of ovariectomized rats and rhesus monkeys primed with estrogen increases uterine weight relative to controls, and also causes increased arteriole number per unit area, dilated blood vessels on the endometrial luminal surface and pronounced endothelial cell proliferation in arterioles and capillaries of the endometrium [9–12]. Implantation bleeding and increased endometrial thickness are observed in relaxin-treated macaque and marmoset monkeys [13, 14], with slightly higher implantation rates following in vitro fertilization and embryo transfer in the macaques [14]. In the context of human physiology, women in a clinical trial for the treatment of scleroderma reported heavier or irregular menstrual bleeding if they were treated with relaxin, possibly due to increased endometrial vascularization [15, 16].

Few studies have investigated the direct effects of relaxin on endometrial remodeling and angiogenesis, although relaxin receptors are expressed in the human endometrium and relaxin is known to modify the expression of numerous key regulatory factors [17–20]. For instance, in rhesus macaques relaxin treatment inhibits endometrial estrogen receptor α (ERα), progesterone receptor A (PRA) and progesterone receptor B (PRB) protein levels [9]. Relaxin also stimulates expression and secretion of the pro-angiogenic factor vascular endothelial growth factor (VEGF) in normal human endometrial stromal and glandular epithelial cells in a dose-dependent manner in vitro [15, 21]. This activation occurs through ERα [22]. Relaxin treatment in macaques has negative impacts on matrix metalloproteinase (MMP) expression causing decreased pro-MMP and an increase in the MMP inhibitor tissue inhibitor of metalloproteinase 1 (TIMP1) [9]. Although these studies provide evidence to support a role for relaxin in uterine vascularization and remodeling in the pre-implantation period, they are limited in their analysis of angiogenesis-related genes. Therefore, the first aim was to undertake a more comprehensive microarray approach using human endometrial stromal (HES) cells treated with relaxin *in vitro* to identify candidate angiogenesis-related genes for further investigation.

The relaxin gene knockout (*Rln*^*-/-*^) mouse provides a model to investigate endogenous relaxin physiology in the pre-implantation period *in vivo*; fetal weights in late pregnancy and pup weight at birth are both significantly reduced, suggesting that mice born to *Rln*^*-/-*^ mice are growth restricted *in utero* [23, 24]. Based on the relaxin treatment effects in rodents and rhesus monkeys [9–14], we predicted that this growth restriction could be due to reduced uterine angiogenesis and remodeling in the uterus of *Rln*^*-/-*^ mice. Therefore, the second aim of this study was to investigate whether or not relaxin deficiency affected uterine expression of representative genes associated with angiogenesis and uterine remodeling, and also blood vessel proliferation in the pre-implantation endometrium (days 1–4 of pregnancy in mice). This time frame was selected so we could focus on the endometrial changes that occur prior to implantation and independently of any influence from the implanting embryo. As there are no data on paracrine relaxin signaling (relaxin and relaxin receptor, *Rxfp1*, expression) in the mouse uterus, a third aim was to demonstrate that relaxin receptors are present in the endometrium and that there is a uterine source of relaxin in early pregnancy.

## Methods

### Animals

All animal experiments were approved by The University of Melbourne Animal Experimental Ethics Committee (AEEC #0911478.1) and conducted in accordance with the Australian Code of Practice and the National Health and Medical Research Council. This study used wild type (*Rln*^*+/+*^) and *Rln*^*-/-*^ mice. Homologous recombination in embryonic stem cells was used to disrupt the relaxin gene by deleting a region essential for biological activity and replacing it with the neomycin transferase gene [25]. *Rln*^*-/-*^ mice were then backcrossed to a C57/BLK6J background and the F14 generation relocated to the School of BioSciences. Genotypes were confirmed by PCR analysis of genomic DNA from ear clips as previously described [25]. Mice were maintained on an automated time cycle of 12 h light/dark at 20 °C, with laboratory chow (Barastock, Pakenham,VIC, Australia) and water available *ad libitum*.

### Identification of RXFP1 in the endometrium of *Rxfp1*^*TG*^ mice

Uterine tissues from non-pregnant transgenic mice with a modified *Rxfp1* gene (*n* = 3) were kindly provided by Prof AI Agoulnik (Florida International University, Miami, FL, USA). The modification is a 4 kb fragment containing parts of exons 15 and 17 that was ligated into a *Sal*I site of the pNTR-LacZ/PGKneo/loxP vector as described by Kamat et al [26]. During homologous integration, the targeting vector was inserted into exon 17 and created a duplication of the 4 kb genomic fragment. Uterine tissues were dissected from *Rxfp1*^*TG*^ mice after anesthetic overdose and were fixed in 2 % paraformaldehyde in 1X phosphate buffered saline/MgCl_2_ for 30 mins at RT. Tissues were then embedded in Tissue-Tek OCT compound (Sakura Finetechnical Co. Ltd), and cryosections were cut at 10 μm and mounted on Superfrost slides. For histological analysis of LacZ expression, tissues sections were processed for X-gal staining using the LacZ Detection Kit for Tissues (InvivoGen) according to the manufacturer’s instructions. Nuclei were then counterstained with 0.1 % nuclear fast red and mounted in aqueous mounting medium. Control tissues were obtained from wild type littermates.

### Effects of relaxin treatment on human endometrial stromal cells in vitro

HES cells were isolated from a collagenase-treated uterus of a pre-menopausal woman who had a hysterectomy for reasons other than uterine disease, with informed consent and approval from the institutional review board [15, 27]. Stromal cells obtained from a normal uterus were cultured in 75 mm^2^ flasks, and fed every two days with DMEM:F12 medium plus 10 % newborn calf serum and 2 mM L-glutamate. These cells have previously been shown to possess high affinity relaxin binding sites [15] and to produce cAMP in response to relaxin [27]. Once confluent, the cells were washed with phosphate buffered saline (PBS) and treated in serum-free DMEM:F12 medium with either vehicle alone or with recombinant human relaxin (rhRLX) (Connetics Corporation, Palo Alto, CA, USA) (10 ng/ml) for 24 h. This concentration and duration was shown to be bioactive in a previous study [15]. Total RNA was isolated using Trizol (Invitrogen) according to manufacturer’s instructions. Total RNA from 8 HES cell cultures treated with vehicle or with rhRLX were pooled to yield 100 μg RNA each. This was sent to Incyte Genomics (Palo Alto) for extraction of PolyA-RNA and subsequent microarray analysis [28]. Briefly, 600 ng of polyA-RNA of each sample was used for reverse transcription and attachment of the appropriate cyanine (Cy) dye. The Cy3- and Cy5-labelled probes were then mixed, and simultaneously hybridized to a single cDNA microarray. Following incubation, the array was washed in 3 consecutive washes of decreasing ionic strength, and scanned in both Cy3 and Cy5 channels using GenePix scanners (Foster City). Incyte GEMtools software was used for image analysis. Background-subtracted element signals are used to calculate Cy3:Cy5 ratios. Default criteria in GEMtools analysis are that the signal-to- background ratio > 2.5 for each element, and that the area for which pixel values are counted is at least 40 % of the element for both signals. The ratio of fluorescent intensities from the two dyes were used to assess relative transcript abundance in the two RNA samples. The minimum detectable fold change for differential expression, according to evaluation of the performance of the Incyte array, is 1.4-fold [29], i.e. expression in mRNA from rhRLX-treated cells 1.4-fold higher than in vehicle-treated cells, or vice versa.

### Effects of relaxin deficiency in early pregnancy

In Study 1, *Rln*^*+/+*^ mice were studied on days 1, 2, 3 and 4 of pregnancy to assess changes in gene expression in the wild type mice (*n* = 8/genotype/day). Based on data from Study 1, we then chose to examine pre-implantation phenotypes in *Rln*^*+/+*^ and *Rln*^*-/-*^ mice only on days 1 and 4 of pregnancy (Study 2, *n* = 8/genotype/day). In Study 3, gene expression alone was examined in an additional group of mice on day 6 of pregnancy (*n* = 7/genotype) to examine if phenotypes persisted after implantation (Additional file 1: Figure S1). Female mice aged 2–4 months were housed with a stud male of matched genotype overnight and then checked for mating plugs the following morning. The presence of a mating plug was an indication of successful mating and was considered day 1 of pregnancy. Four hours prior to tissue collection, mice received a 500 μl i.p. injection of bromodeoxyuridine (BrdU; 2 mg/ml, Sigma-Aldrich Pty Ltd), enabling later visualization of proliferating cells by immunohistochemistry. After isofluorane anesthesia, blood was collected by cardiac puncture and the plasma stored at -80 °C. After cervical dislocation, the uterine tissues were removed with half fixed in 10 % neutral buffered formalin overnight before processing for paraffin sections, and the other half stored at -80 °C for gene expression analysis.

### Analysis of gene expression

Total RNA was isolated from whole uteri using Trizol reagent (Invitrogen) according to the manufacturer’s instructions. The concentration of RNA was determined using a NanoDrop ND100 Spectrophotometer (NanoDrop Technologies Inc) with A260:A280 absorbance ratios of > 2.0 indicating sufficiently pure RNA for PCR analysis. First strand cDNA synthesis used 1 μg total RNA in a 20 μL reaction, using random hexamers (50 ng/μL), 10 mM dNTP and 200 U SuperScript III (Invitrogen). First-strand cDNA synthesis for all samples were performed simultaneously at 25 °C for 10 min, 50 °C for 50 min and 85 °C for 5 min. Quantitative reverse-transcription polymerase chain reaction (quantitative PCR) was performed to assess the relative expression of relaxin (*Rln*) and its receptor *Rxfp1* and key genes chosen to represent angiogenesis (total VEGFA (*VegfA*), VEGF receptor 2 (*Vegfr2*)), steroid receptors (estrogen receptor (*ER)-α* (*Esr1*), ER-β (*Esr2*), progesterone receptor (*Pgr*)), tissue remodeling (matrix metalloproteinase 14 (*Mmp14*), *Timp3*)), and other key genes identified in our microarray analysis (ankryn repeat domain 37 (*Ankrd37*), egl-9 family hypoxia-inducible factor 1 (*Egln1*), hepatocyte growth factor (*Hgf*), hypoxia inducible factor 1α (*Hif1α*))*.* In addition to these genes investigated pre-implantation, interleukin 1β (*Il1β*) was also investigated post-implantation (Additional file 2: Figure S2). Forward and reverse primers and 6-carboxy fluorescein (FAM)-labeled TaqMan probes specific for mouse genes were designed to span an intron/exon junction (Biosearch Technologies, Inc). Primer sequences are shown in Additional file 3: Table S1. All quantitative PCR reactions for each gene were performed on one plate, in 10 μl or 20 μl 96-well reactions containing SensiFAST Probe Lo-Rox (BioRad) and performed in the ViiA™7 Real-Time PCR System (Life Technologies). Recent data suggest that glyceraldehyde 3-phosphate dehydrogenase (*Gapdh*) and 18 s ribosomal RNA (*18 s*) expression are upregulated in the rodent uterus in response to progesterone and estrogen [30, 31]. The same pattern of *18 s* mRNA expression was observed in this study so we investigated peptidylprolyl isomerase A (*Ppia*), succinate dehydrogenase complex, subunit A (*Sdha*) and TATA box binding protein (*Tbp*) as reference genes; however, these were also significantly increased from days 1 to 4 of pregnancy (Additional file 2: Figure S2). Therefore we used the mean C_T_ value of the *Rln*^*+/+*^ mice on day 1 of pregnancy as the internal calibrator and subtracted this from the mean gene of interest triplicate C_T_ value (Ratio_(test/calibrator)_ = 2^ΔCT^, where ΔC_T_ = C_T(calibrator)_ − C_T(test)_) as has been described previously [30, 32]. These “normalized” data (ΔC_T_) were presented as fold induction relative to day 1 of pregnancy in the *Rln*^*+/+*^ mice. The mean C_T_ values and range C_T_ values for each gene are presented in Additional file 3: Table S2.

The presence or absence of relaxin (*Rln*) and *Rxfp1* gene transcripts in the uterus and ovaries were assessed by reverse transcriptase polymerase chain reaction (RT-PCR) in the GeneAmp PCR system 2700 (Applied Biosystems). Reaction mixes consisted of GoTaq Green (Promega), mouse-specific oligonucleotide primers (100 ng/μl) designed to span an intron/exon junction (Additional file 3: Table S3) and 1 μl cDNA. Ovary RNA was extracted as described above.

### Immunohistochemistry

As the study focused on pre-implantation vascular remodeling, we used immunohistochemistry to examine endometrial endothelial cell proliferation as a marker of endometrial angiogenesis. We used a dual immunohistochemistry protocol with antibodies against CD31 (pan-endothelial cell marker) and BrdU (labels proliferating cells). After dewaxing and rehydration, sections (5 μm) were incubated with 0.1 % (1 mg/ml) pepsin in 3 % acetic acid for 10 min at 37 °C, followed by serum free protein block (Dako) for 15 min to prevent non-specific binding. Sections were incubated with rat monoclonal anti-mouse CD31 (5 mg/ml in 1 % bovine serum albumin in PBS (BSA/PBS) (BD PharMingen)) overnight at 4 °C. Sections were then incubated for 1 h at room temperature in biotinylated goat anti-rat IgG (1:200 in 1 % BSA/PBS, Chemicon), followed by incubation in alkaline phosphatase conjugated streptavidin (Dako) for 15 min. CD31-positive immunostaining was visualized using the vector blue alkaline phosphatase chromagen (10 min, Vector Laboratories). Sections were then incubated in 0.1 M HCl for 45 min at room temperature, followed by a further incubation 3 % H_2_O_2_ in PBS for 10 min at room temperature to quench endogenous peroxidase. Serum free protein block was applied for a further 15 min. Sections were incubated for 1 h at room temperature in monoclonal sheep anti-BrdU (8 μg/ml in 1 % BSA/PBS, Abcam). This was followed by a further incubation for 1 h at room temperature in donkey anti-sheep IgG (4 μg/mL in 1 % BSA/PBS, Jackson Immuno Research Laboratories). Sections were then incubated in streptavidin horseradish peroxidase (Dako) for 15 min, followed by 5 min in DAB chromagen (3,30-diaminobenzidine, Sigma-Aldrich) to visualize BrdU immunostaining. Each slide contained a negative isotope matched control section that was prepared by replacing the CD31 primary antibody with rat IgG2a (BD Biosciences) and the BrdU primary antibody with sheep IgG (Sigma-Aldrich) at the same concentrations as that of the primary antibodies. For the main analysis, we analyzed one section per uterus (*n* = 7/group) taken from the mid-point between the cervix and ovaries. The number of proliferative endothelial cells and the number of blood vessel profiles present in the endometrial tissue on each section were counted. Data are presented as the proportion of vessel profiles containing one or more proliferating endothelial cells, and as the number of proliferating endothelial cells per mm^2^ (identified using a × 40 objective lens).

### Steroid analysis

Plasma progesterone and corticosterone concentrations were measured by triple quadrupole mass spectrometry (Eric Thorstensen, Liggins Institute, University of Auckland, Auckland, New Zealand). Estrogen could not be measured as there was insufficient plasma for accurate detection. An internal standard solution (60 ng mL^-1^ corticosterone-d8 and 0.2 ng mL^-1^ estradiol-d3 in water) was added to each plasma sample (100 μL standard to 200 μL plasma). Steroids were then extracted using 1 mL of ethyl acetate (Merck). After removal of the organic supernatant to a clean tube, samples were dried by vacuum concentration (Savant SC250EXP, Thermo Scientific), resuspended in 60 μL of mobile phase (45 % methanol and 55 % water) and transferred to HPLC injector vials. Samples (12 μL) were injected onto an HPLC mass spectrometer system consisting of an Accela MS pump and autosampler followed by an Ion Max APCI source on a Finnigan TSQ Quantum Ultra AM triple quadrupole mass spectrometer all controlled by Finnigan Xcalibur software (Thermo Electron Corporation). The mobile phase was a gradient of methanol and water, flowing at 400 μL.min^-1^ through a Hypersil Gold 1.9 μm C18 50 ×2.0 mm column at 40 °C (Thermo Fisher Scientific). Retention time was 6.2 min for progesterone and 2.3 min for corticosterone. Ionization was in positive mode for progesterone, Q2 had 1.2 mTorr of argon. The mass transitions followed were: progesterone 315.5 → 109.2 at 22 V and corticosterone 347.2 → 121.0 at 27 V.

### Statistical analysis

Proliferating endothelial cell number and circulating steroid hormone concentrations were assessed by two-way ANOVA (genotype v day of pregnancy) and Tukey post-hoc tests (SPSS Inc.). Changes in gene expression during early pregnancy in the *Rln*^*+/+*^ mice were assessed by one-way ANOVA (day of pregnancy) and Tukey post-hoc tests. Two-way ANOVA with genotype and day of pregnancy as main effects assessed any interaction between genotype and day of pregnancy in the *Rln*^*+/+*^ and *Rln*^*-/-*^ mice followed by Tukey post-hoc tests to compare means of all groups. T-tests were used to examine gene expression differences between *Rln*^*+/+*^ and *Rln*^*-/-*^ mice on day 6 of pregnancy. If not normally distributed, raw data were log transformed and non-parametric analysis was performed using Mann-Whitney with Bonferroni correction. All figures are graphed using the untransformed fold change data.

## Results

### Relaxin signaling in early pregnancy

**Fig. 1:**
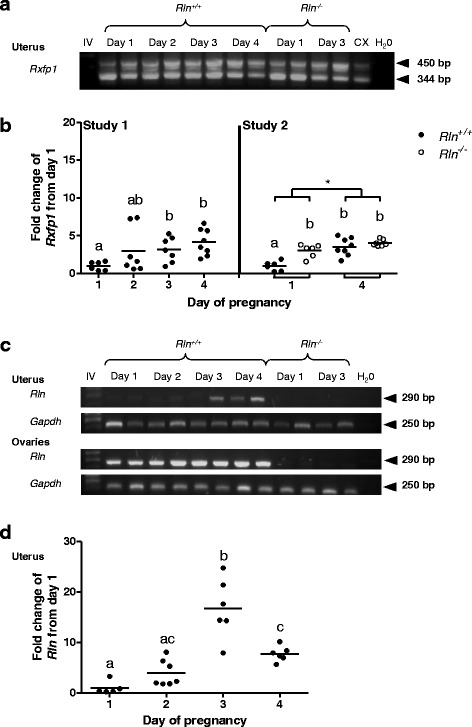
**a** Reverse transcriptase PCR (RT-PCR) expression of *Rxfp1* in the uterus of wild type (*Rln* ^*+/+*^) and relaxin deficient (*Rln* ^*-/-*^) mice in early pregnancy. The positive control for *Rxfp1* was the cervix (CX). Note the two RXFP1 isoforms. The negative control was water (H_2_O). IV = Hyperladder OV. **b** Quantitative PCR analysis of *Rxfp1* mRNA in the uterus of *Rln* ^*+/+*^ mice from days 1 to 4 of pregnancy (Study 1), and in *Rln* ^*+/+*^ and *Rln* ^*-/-*^ mice on days 1 and 4 (Study 2) (*n* = 6–8). **c** RT-PCR expression of relaxin (*Rln)* and *Gapdh* in the uterus and ovaries of *Rln* ^*+/+*^ and *Rln* ^*-/-*^ mice in early pregnancy. **d** Quantitative PCR analysis of *Rln* mRNA in the uterus of *Rln* ^*+/+*^ mice from days 1 to 4 of pregnancy. Expression is shown as the fold change compared with the mean gene expression in *Rln* ^*+/+*^ mice on day 1 of pregnancy. Horizontal bars indicate mean values. Groups within each study that do not share a letter are significantly different from one another, *denotes a significant difference between day 1 and 4 of pregnancy in study 2, significance is *p* < 0.05

Reverse transcriptase PCR confirmed the presence of two gene transcripts for *Rxfp1* representing both isoforms in the uterus of both *Rln*^*+/+*^ and *Rln*^*-/-*^ mice in early pregnancy (Fig. 1a) (raw C_T_ values in Additional file 3: Table S2). There was a significant (*p* = 0.006) increase in *Rxfp1* mRNA in *Rln*^*+/+*^ mice on days 3 and 4 of pregnancy compared with day 1 (Fig. 1b). A comparison between genotypes revealed a significant (*p* = 0.003) increase in *Rxfp1* expression on day 1 in *Rln*^*-/-*^ mice compared to day 1 in *Rln*^*+/+*^ mice but expression on day 4 of pregnancy was similar between *Rln*^*+/+*^ and *Rln*^*-/-*^ mice (Fig. 1b). *Rln* mRNA was detected at all stages in the ovaries of the wild type mice (Fig. 1c). *Rln* mRNA was also detected in whole uterus, with significantly increased expression on days 3 and 4 of pregnancy relative to day 1 (*p* < 0.05) (Fig. 1c and d).

**Fig. 2:**
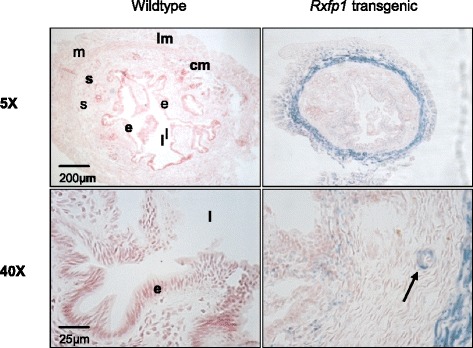
RXFP1 receptor localization in tissues in the uterus of non-pregnant wild type and *Rxfp1*-transgenic mice. Strong positive staining for β-galactosidase activity (*blue*) indicates RXFP1. s = stroma; l = lumen; e = luminal epithelium; cm = circular layer of myometrium; lm = longitudinal layer of myometrium. Magnifications are indicated on the *left*

Strong positive staining for *Rxfp1*-specific β-galactosidase activity was identified in the myometrium of *Rxfp1*^*TG*^ mice, particularly in the inner circular muscle layer (Fig. 2). Upon closer examination of the endometrium, positive staining for *Rxfp1*-specific β-galactosidase activity was seen in the stroma towards the luminal epithelium. The negative control used in this study was the uterus of a wild type mouse, which showed no endogenous or background β-galactosidase activity (Fig. 2). This confirmed that the β-galactosidase activity observed in the *Rxfp1*^*TG*^ mouse uterus was specific and represents localization of *Rxfp1*.

### Effects of relaxin on angiogenesis-related gene expression in human endometrial stromal cells

A total of 321 genes were upregulated (by 1.4-fold or more) in HES cells incubated with rhRLX for 24 h, whereas 203 genes were down-regulated by at least 30 %. 64 well characterized genes increased with relaxin treatment (Table 1), of which glycerol kinase showed the largest increase (2-fold). A number of genes whose expression is associated with regulation of angiogenesis were among those modulated by rhRLX. *Timp3* (1.9-fold), *Hgf*, (1.5-fold) *Hif1α* (1.5-fold) and angiogenin (1.4-fold) were increased. The expression of VEGF was analyzed as 1.2-fold higher in relaxin-treated cells, within the noise of the assay. In contrast, VEGF-D and CD36 were decreased by 30 %. CD36 thought to be an inhibitor of angiogenesis [33] was down-regulated by 30 % after rhRLX treatment.

**Table 1:** Selected genes modulated by human recombinant relaxin treatment of human endometrial stromal cells in vitro

Category	Gene	Accession number	Fold-change
Angiogenesis related	Tissue inhibitor of metalloproteinase-3	NM_000362	+1.9
	Hypoxia-inducible factor 1, alpha subunit	NM_001530	+1.5
	Hepatocyte growth factor	NM_000601	+1.5
	Angiogenin	NM_001145	+1.4
	Vascular endothelial growth factor A	NM_001025366	+1.2
	Vascular endothelial growth factor D	NM_004469	- 0.3
Angiogenesis inhibitors	CD36	NM_001001548	- 0.3
Hemostasis/menses correlated	Matrix metalloproteinase-7	NM_002423	- 0.4
	Tissue plasminogen activator	NM_000930	- 0.3
Secretory phase expression	Interleukin 1 receptor-like 1	NM_003856	+1.5
	CD44	NM_000610	- 0.3
Proliferative phase expression	Tenascin C	NM_002160	- 0.4
	Androgen receptor	NM_000044	- 0.3
Renin-angiotensin system	Renin	NM_000537	+1.4
	Angiotensin II receptor 1	NM_000685	+1.4
	Angiotensin II receptor 2	NM_000686	- 0.3
Growth factors/cytokines	Insulin-like growth factor binding protein 4	NM_001552	+1.4
	Insulin-like growth factor 1	NM_001111284	+1.4
	Osteoclast stimulating factor 1	NM_012383	+1.4
	Amphiregulin	NM_001657	+1.4
	Connective tissue growth factor	NM_001901	- 0.3
Structural proteins	Dystrophin	NM_004011	+1.6
	Claudin 10	NM_006984	+1.5
	Tetraspan 3	NM_005724	+1.4
	Myosin X	NM_012334	+1.4
	Contactin 1	NM_001843	+1.4
	Keratocan	NM_007035	- 0.5
	Matrin 3	NM_199189	- 0.3
	Sorcin	NM_003130	- 0.3
	Cadherin-2	NM_001792	- 0.3
	Tight junction protein 1	NM_003257	- 0.3
Extracellular matrix	Fibronectin	NM_212482	- 0.3
	Nidogen (entactin)	NM_002805	- 0.3
Metabolism	Glycerol kinase	NM_203391	+2.0
	Acyl-Coenzyme A dehydrogenase	NM_000016	+1.6
	Ceruloplasm	NM_000096	+1.4
	Transferrin	NM_001063	+1.4
Enzymes/inhibitors	Heparin cofactor II	NM_000185	+1.8
	Serine protease inhibitor	NM_001127698	+1.5
	Carboxypeptidase B2	NM_001872	+1.5
	Aminoacylase 1	NM_000666	+1.4
	Cathepsin B	NM_001908	- 0.3
Transcription factors	Kruppel-like factor-1	NM_006563	+1.5
	Eukaryotic translation termination factor 1	NM_004730	+1.5
	Eukaryotic translation initiation factor 3	NM_003758	+1.5
	Transactivator jun-B	NM_002229	+1.4
Protein kinases	Mitogen-activated protein kinase 4	NM_002747	+1.5
	Protein kinase, cAMP-dependent regulatory	NM_002734	+1.4
	Calcium/calmodium protein kinase I	NM_003656	+1.4
	Calcium/calmodium protein kinase II	NM_172127	+1.4
	Protein kinase, cAMP-dependent catalytic	NM_002731	- 0.4
	Mitogen-activated protein kinase 1	NM_002745	- 0.3
	Cyclin G1	NM_004060	- 0.3
Receptors	Retinal G-protein coupled receptor	NM_002921	+1.5
	Hyaluronan-mediated motility receptor	NM_001142556	+1.4
	Vasoactive intestinal peptide receptor 2	NM_003382	+1.4
	Activin A receptor IB	NM_004302	- 0.3
	Chemokine receptor 1	NM_001295	- 0.3
Others	Lacrimal proline-rich protein	NM_007244	+1.6
	Trefoil factor-1	NM_003225	+1.6
	Amyloid precursor protein binding protein-2	NM_006380	+1.5
	Neurogenic differentiation 1	NM_002500	+1.5
	Annexin A3	NM_005139	+1.5
	Basonuclin 1	NM_001717	- 0.5

### Gene expression in the uterus of early pregnant Rln^+/+^ and Rln^-/-^ mice

**Fig. 3:**
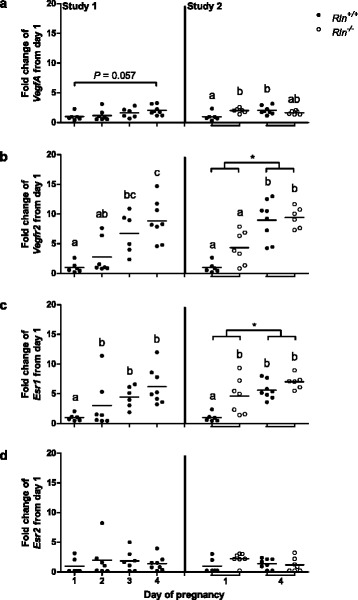
Expression of (**a**) *VegfA* total, (**b**) *Vegfr2*, (**c**) *Esr1* and (**d**) *Esr2* in the uterus of *Rln* ^*+/+*^ mice on days 1 to 4 of pregnancy (Study 1), and in *Rln* ^*+/+*^ and *Rln* ^*-/-*^ mice on days 1 and 4 of pregnancy (Study 2) (*n* = 6–8). Horizontal bars indicate mean values. Groups within each study that do not share a letter are significantly different from one another, *denotes a significant difference between day 1 and 4 of pregnancy in study 2, significance is *p* < 0.05

Consistent with prior studies [34], there was a modest increase in *VegfA* on day 4 of pregnancy compared with day 1 in the wild type mice in study 2 (*p* = 0.012) (Fig. 3a). A comparison between genotypes demonstrated a significant (*p* = 0.026) increase in *VegfA* on day 1 of pregnancy in the *Rln*^*-/-*^ mice compared with the same time point in *Rln*^*+/+*^ mice. Similarly, there was a significant (Day 3: *p* = 0.006; Day 4: *p* = 0.002) increase in *Vegfr2* on days 3 and 4 of pregnancy in *Rln*^*+/+*^ mice compared with day 1 but there was no effect of genotype (Fig. 3b).

Because relaxin is known to modulate steroid receptor expression [9], *Esr1* and *Esr2* transcripts were examined (*Pgr* was also examined, see below). *Esr1* but not *Esr2* was significantly (Day 2: *p* = 0.01; Day 3: *p* = 0.002; Day 4: *p* = 0.004) increased in *Rln*^*+/+*^ mice on days 2, 3 and 4 of pregnancy. Only *Esr1* expression was significantly (pregnancy: *p* ≤ 0.001; genotype: *p* = 0.002) affected by both pregnancy and genotype (Fig. 3c and d).

**Fig. 4:**
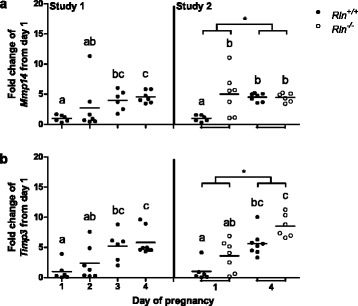
Expression of extracellular matrix remodeling genes (**a**) *Mmp14* and (**b**) *Timp3* in the uterus of *Rln* ^*+/+*^ mice from days 1 to 4 of pregnancy (Study 1) and in *Rln* ^*+/+*^ and *Rln* ^*-/-*^ mice on days 1 and 4 of pregnancy (Study 2) (*n* = 6–8). Horizontal bars indicate mean values. Groups within each study that do not share a letter are significantly different from one another, *denotes a significant difference between day 1 and 4 of pregnancy in study 2, significance is *p* < 0.05

*Mmp14* and *Timp3* were also significantly (*Mmp14*: Day 3: *p* = 0.012; Day 4: *p* = 0.003, *Timp3*: Day 3: *p* = 0.019; Day 4: *p* = 0.004) upregulated on days 3 and 4 compared with day 1 in pregnant *Rln*^*+/+*^ mice (Fig. 4). Interestingly, *Mmp14* was significantly (*p* = 0.001) higher in *Rln*^*-/-*^ mice on day 1 of pregnancy compared with the *Rln*^*+/+*^ counterpart, whereas there was no significant effect of genotype on *Timp3* expression. However *Timp3* was significantly (pregnancy: *p* ≤ 0.001) higher on day 4 of pregnancy compared with day 1 in both genotypes in study 2.

**Fig. 5:**
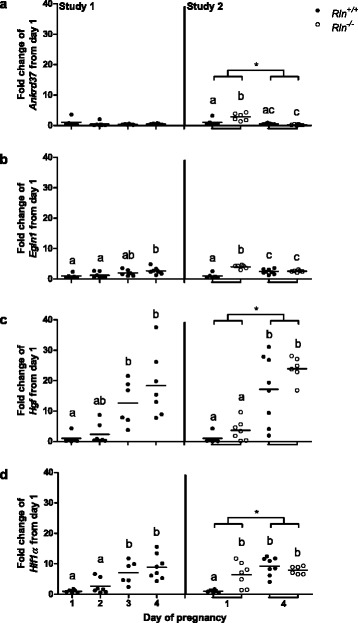
Expression of (**a**) *Ankrd31*, (**b**) *Egln1*, (**c**) *Hgf* and (**d**) *Hif1α* in the uterus of *Rln* ^*+/+*^ mice from days 1 to 4 of pregnancy (Study 1) and in *Rln* ^*+/+*^ and *Rln* ^*-/-*^ mice on days 1 and 4 of pregnancy (Study 2) (*n* = 6–8). Horizontal bars indicate mean values. Groups within each study that do not share a letter are significantly different from one another, *denotes a significant difference between day 1 and 4 of pregnancy in study 2, significance is *p* < 0.05

Based on the data from the microarray study of HES cells (Table 1), we analyzed four other genes that were altered by relaxin treatment. In wild type mice, there was no significant increase in *Ankrd37* from days 1 to day 4 of pregnancy (Fig. 5a). A comparison of genotypes demonstrated a significant (*p* = 0.011) increase in *Ankrd37* on day 1 in the *Rln*^*-/-*^ mice compared with *Rln*^*+/+*^. *Egln1* was significantly (*p* = 0.015) upregulated on day 4 of pregnancy in *Rln*^*+/+*^ mice compared to days 1 and 2 (Fig. 5b). There was also a significant (*p* ≤ 0.001) difference between the genotypes on day 1. Both *Hgf* and *Hif1α* were significantly (*p* < 0.05) increased on days 3 and 4 in the wild type mice compared to day 1 of pregnancy (Fig. 5c and d). *Hif1α* expression was significantly (*p* = 0.008) upregulated in the *Rln*^*-/-*^ mice on day 1 compared with *Rln*^*+/+*^ but there was no difference in *Hgf* between the genotypes.

We also examined expression of the genes noted above in uteri from day 6 pregnant mice, but no significant differences were observed between *Rln*^+/+^ and *Rln*^-/-^ mice (Additional file 1: Figure S1).

### Pre-implantation endometrial angiogenesis

**Fig. 6:**
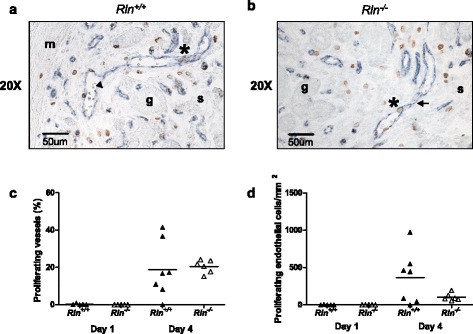
Representative photomicrograph of CD31 (*blue*, pan-endothelial cell marker) and BrdU (*brown*, proliferating cell marker) immunostaining in the endometrium of a (**a**) wild type (*Rln* ^*+/+*^) and (**b**) relaxin deficient (*Rln* ^*-/-*^) mice on day 4 of pregnancy. *Black arrow* indicates blood vessel profile; * = proliferating endothelial cells; g = gland; s = stroma; m = myometrium. **c** Percentage of vessel profiles containing proliferating endothelial cells (%) and the (**d**) number of proliferating endothelial cells per mm^2^ in the endometrium of *Rln* ^*+/+*^ and *Rln* ^*-/-*^ mice on days 1 and 4 of pregnancy (*n* = 5–7). Horizontal lines indicate mean values. Each triangle represents data from an individual mouse

Endothelial cell proliferation occurred in the endometrium on day 4 but not day 1 of pregnancy in wild type mice, consistent with a previous report [6]. Proliferation in the *Rln*^*-/-*^ mice also occurred on day 4 (Fig. 6). However, there was no significant difference in the percentage of vessel profiles containing proliferating endothelial cells or the number of proliferating endothelial cells in the endometrium per mm^2^ between the genotypes. Additionally, there was no difference in the number of vessels per mm^2^ (data not shown).

### Plasma progesterone and corticosterone concentrations

**Fig. 7:**
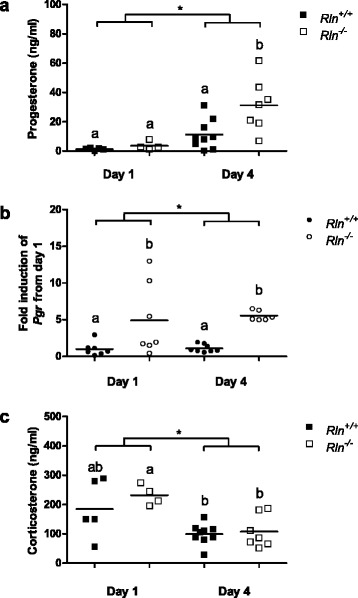
**a** Plasma progesterone concentrations, (**b**) expression of progesterone receptor (*Pgr*) and (**c**) plasma corticosterone concentration in wild type (*Rln* ^*+/+*^) and relaxin deficient (*Rln* ^*-/-*^) mice on days 1 and 4 of pregnancy (*n* = 4–9). Horizontal lines indicate the mean. Groups within each study that do not share a letter are significantly different from one another, *denotes a significant difference between day 1 and 4 of pregnancy in study 2, significance is *p* < 0.05

As seen in previous studies [6], there was a significant (*p* ≤ 0.001) effect of day of pregnancy on circulating progesterone levels in the wild type and relaxin deficient mice from day 1 to day 4 of pregnancy (Fig. 7a). Plasma progesterone concentrations were significantly (*p* = 0.013) different between the two genotypes on day 4, with an increase in progesterone in the *Rln*^*-/-*^ mice relative to the *Rln*^*+/+*^ mice. There was a significant (Day 1: *p* = 0.017; Day 4: *p* = 0.005) increase in *Pgr* in *Rln*^*-/-*^ mice compared to *Rln*^*+/+*^ mice on days 1 and 4 of pregnancy (Fig. 7b). Circulating corticosterone levels were significantly (*p* = 0.003) higher on day 1 compared with day 4 of pregnancy in both *Rln*^*+/+*^ and *Rln*^*-/-*^ mice (Fig. 7c) but there was no difference between the genotypes.

## Discussion

The aims of this study were to confirm expression of relaxin and its receptor RXFP1 in the whole uterus of early pregnant mice prior to implantation and examine the effects of relaxin deficiency on endometrial angiogenesis and the expression of key angiogenesis-related and extracellular matrix remodeling genes in the pre-implantation period. Because fetal growth is compromised in *Rln*^-/-^ mice, and relaxin is thought to play a role in vascular growth in the endometrium in primates, our prediction was that pre-implantation angiogenesis in the endometrium would be compromised in *Rln*^*-/-*^ mice. In fact, we observed that blood vessel proliferation was not different in *Rln*^*-/-*^ mice compared to wild types before implantation. Unexpectedly, this was despite a general increase in the expression of many angiogenesis-related and other remodeling genes in the uteri of *Rln*^*-/-*^ mice compared to wild type animals, particularly on day 1 of pregnancy. Relaxin deficiency was also associated with an increase in circulating progesterone levels and progesterone receptor mRNA expression. Overall, our data demonstrate phenotypic changes in the pre-implantation uterus of *Rln*^*-/-*^ mice that include enhanced expression of steroid receptors, angiogenesis and matrix remodeling-related genes, but without the predicted impact on endometrial angiogenesis.

Circulating relaxin is undetectable in mice in early pregnancy [35] but relaxin gene transcripts were detected in the ovaries and uterus from day 1 of pregnancy. The surprising finding was the high expression of relaxin on day 3 of pregnancy for which we have no explanation. Previous studies have shown relaxin gene expression in the uterus of early pregnant pigs [36] and in HES cells in culture [21]. These data provide good evidence that relaxin is locally produced and can act in a paracrine fashion in the uterus. It was also important to demonstrate *Rxfp1* expression in the uterus of mice in early pregnancy. *Rxfp1* increased between days 1 and 4 of pregnancy in both *Rln*^*+/+*^ and *Rln*^-/-^ mice. In fact, *Rxfp1* was higher in *Rln*^-/-^ mice at the start of pregnancy. Increased *Rxfp1* expression has also been shown in the cervix and vagina of *Rln*^*-/-*^ mice [37] but not in the myometrium in late pregnancy [38]. This suggests differential regulatory effects of the relaxin ligand on its own receptors dependent on tissue and stage of pregnancy [39].

LacZ staining demonstrated that RXFP1 was localized predominantly in the inner circular layer of the myometrium, but also in the endometrial stroma similar to that shown by Kamat et al [26]. However, this observation was made in non-pregnant mice, so it is possible that the pattern of RXFP1 staining may differ in early pregnant mice, likely intensified in the endometrium. Several studies have reported high expression of immunoreactive RXFP1 in endometrial stromal cells in uterine samples from women during the menstrual cycle [17, 18] but this was not substantiated in later studies in which RXFP1 mRNA and relaxin binding sites were predominantly found in glandular and luminal epithelial cells [19]. No studies to date have assessed RXFP1 in early pregnancy in relation to angiogenesis. In women, there was an increase in RXFP1 in endometrial biopsies collected during the secretory phase relative to those from the proliferative phase [19]. It was not established if changes in RXFP1 occurred in stromal, epithelial or endothelial cells within the endometrium. However, Kohsaka et al [40] identified relaxin binding sites in epithelial cells, smooth muscle cells, and blood vessels in the cervix, vagina and uterus in tissues obtained from women after hysterectomy. RXFP1 has been localized specifically to endothelial cells [41, 42] in systemic and uterine blood vessels, but it remains to be demonstrated if relaxin receptors are expressed in endometrial endothelial cells in early pregnancy.

Because HES cells have previously been shown to express high affinity relaxin binding sites [15] and to secrete cAMP [27] and VEGF [15] in response to relaxin, these cells were used to screen for other potentially relevant genes upregulated by relaxin. Our microarray analysis of gene transcripts altered by relaxin in HES cells revealed a number of genes whose expression is associated with stimulation of angiogenesis, including *Timp3*, *Hgf*, and *Hifiα*. These and other genes known to modulate steroid responsiveness, angiogenesis and extracellular remodeling, including *Esr1*, *VegfA, Vegfr2, Pgr*, *Elgn1*, *Mmp14* and *Ankrd37*, were investigated in the mouse uterus by quantitative analysis. Genes including *Esr1*, *Hgf*, *Hif1α*, *Timp3*, *VegfA*, *Vegfr2* and *Pgr* were all upregulated by day 4 of pregnancy in the uterus of *Rln*^*+/+*^ mice relative to day 1. This correlates well with previously published data [34, 43–46]. In addition, two other novel genes were upregulated in the uterus of early pregnancy, *Egln1* and *Mmp14*. The latter remodels the ECM by activating other MMPs including MMP2 [47].

Fewer genes were analyzed in the *in vivo* study in mice; surprisingly, there was a significant increase in most of the genes examined on day 1 of pregnancy in the *Rln*^*-/-*^ mice. Most notably, there was a large increase in *Pgr* and *Esr1*, but not *Esr2,* as well as an increase in circulating progesterone on day 4. This contrasts with previous work that demonstrated increases in *Esr2*, but not *Esr1*, in the cervix and vagina of late pregnant *Rln*^*-/-*^ mice [37]. These differences may simply be due to tissue-specific expression or because we investigated the uterus prior to implantation, a time when the steroid hormone profile is very different to that later in pregnancy. Treatment of ovariectomized rhesus monkeys with relaxin reduces endometrial progesterone receptor isoforms A and B, and ERα, without effecting ERβ [9]. These data support our findings in the *Rln*^-/-^ mice, and indicate a role for endogenous relaxin in the regulation of these receptors. Additionally, this accelerated increase in *Pgr* in the *Rln*^*-/-*^ mice could indirectly mediate the increased expression of the angiogenesis and extracellular matrix remodeling related genes on day 1 of pregnancy; further, we suggest that increased plasma progesterone levels, with increased expression of progesterone receptors, may be sufficient to compensate for the lack of relaxin in the stimulation of angiogenesis.

Another surprising finding was the increased *VegfA* expression in *Rln*^*-/-*^ mice on day 1 of pregnancy. Relaxin stimulates expression and secretion of VEGF in human endometrial stromal and glandular epithelial cells in a dose-dependent manner in vitro [15, 21]. Therefore, we had predicted that in the absence of relaxin in early pregnancy, *VegfA* would be compromised. However, progesterone mediates its angiogenic effects partly through VEGF so the enhanced progesterone/*Pgr* expression on day 1 of pregnancy in *Rln*^*-/-*^ mice could explain the increase in *VegfA* expression.

Similarly, given the rhRLX-induced increase in *Hgf* and *Hif1α* in the human microarray study, we anticipated that expression of these genes would be decreased in the uterus of the *Rln*^-/-^ mice. This was not the case. The significance of the increased expression of *VegfA*, *Esr1*, *Egln1*, *Hif1α*, *Mmp14* and *Ankrd37* in the *Rln*^*-/-*^ mice on day 1 of pregnancy is unknown. One possibility is that in the absence of relaxin, the estrous cycle is modified, with a shorter follicular phase and more rapid onset of estrus. Although our mice were synchronized around estrus, they could mate any time between 4 pm and 8 am when they were first checked for mating plugs. If the *Rln*^-/-^ mice had shorter estrous cycles, they could mate up to 12 h before *Rln*^+/+^ mice, and therefore post-coital stimuli that trigger changes in the uterine environment would be accelerated in *Rln*^-/-^ mice. Of interest, circulating corticosterone levels were significantly elevated in both genotypes on day 1 of pregnancy relative to day 4, suggesting a possible stress response to mating and/or human contact post-coitus.

There are two noteworthy limitations in our study. First, our gene analysis used the whole uterus because it was not possible to separate the endometrium from the myometrium in the mouse uterus in early pregnancy and yield sufficient quantities of high quality RNA. We acknowledge that the homogenized uteri contain both endometrium and myometrium, and that there are spatiotemporal differences in the genes analyzed. Thus it is possible that relaxin deficiency could affect spatiotemporal gene expression. This could only be assessed through *in situ* hybridization. Second, we used HES cells in the microarray analysis to identify genes targeted by relaxin. Patterns of relaxin secretion vary in humans and rodents, with relaxin peaking in the first trimester in humans [48] and increasing throughout pregnancy in rodents [35, 49]. Furthermore, endometrial angiogenesis occurs in women before the arrival of the blastocyst and primarily occurs in mice after implantation. Therefore the role of relaxin in these two species could be quite different, and the pre-implantation uterus may not be a substantial relaxin target in mice. Importantly, we would argue that relaxin-deficient mice are not an appropriate animal model to further investigate the role of relaxin in the pre-implantation uterus in humans.

## Conclusion

In conclusion, we have demonstrated that relaxin deficiency in mice results in phenotypic changes in the pre-implantation uterus including differential expression of steroid receptor and angiogenesis/remodeling related genes and increased circulating progesterone. Consistent with previously published data, there was an increase in endometrial endothelial cell proliferation by day 4 of pregnancy in the *Rln*^*+/+*^ mice [6]; this was also seen in the *Rln*^*-/-*^ mice. However, there was no significant decrease in endometrial angiogenesis in the relaxin deficient mice. Nor was there accelerated blood vessel proliferation on day 1 of pregnancy in *Rln*^*-/-*^ mice despite the increased expression of angiogenesis-related genes. Therefore, we conclude that endogenous relaxin does not play a major role in pre-implantation angiogenesis in the mouse uterus.

## Additional files

Additional file 1: Figure S1. Expression of (a) *Rxfp1*, (b) *Il1β*, (c) *VegfA* total, (d) *Vegfr2*, (e) *Esr1*, (f) *Esr2*, (g) *Mmp14* and (h) *Timp3* in the uterus of *Rln* ^*+/+*^ and *Rln* ^*-/-*^ mice on day 6 of pregnancy (Study 3, *n* = 6–7). Horizontal bars indicate mean values. (PDF 35 kb) Additional file 2: Figure S2. Expression of (a) *18 s*, (b) *PpiA*, (c) *Sdha* and (d) *Tbp* in the uterus of *Rln* ^*+/+*^ mice on days 1 to 4 of pregnancy (*n* = 6–8). Horizontal bars indicate mean values. Groups that do not share a letter are significantly different from one another (*p* < 0.05). (PDF 37 kb) Additional file 3: Table S1. Primer and probe sequences for the quantitative amplification of murine genes. **Table S2.** Mean C_T_ values and the range of C_T_ values for each gene analyzed by qPCR in the uterus of wildtype (*Rln* ^*+/+*^) and relaxin deficient (*Rln* ^*-/-*^) mice (*n* = 6–8). **Table S3.** Primer sequences for the RT-PCR amplification of murine genes. (DOC 111 kb)

## Abbreviations

*Ankrd37*: Ankryn repeat domain 37 gene
*Egln1*: E gl-9 family hypoxia-inducible factor 1 gene
*ERα*: Estrogen receptor alpha gene
*ERβ*: Estrogen receptor beta gene
HES: Normal human endometrial stromal cells
*Hgf*: Hepatocyte growth factor gene
*Hif1α*: Hypoxia inducible factor 1 alpha gene
*Il1β*: Interleukin 1 beta gene
*Mmp14*: Matrix metalloproteinase 14 gene
*Pgr*: Progesterone receptor gene
rhRLX: Recombinant human relaxin
*Rln*: Relaxin gene
*Rln*^-/-^: Relaxin deficient mice
*Rln*^+/+^: Wild type mice
*Rxfp1*: Relaxin receptor gene
*Timp3*: Tissue inhibitor of metalloproteinase 3 gene
*VegfA*: Vascular endothelial growth factor A gene
*Vegfr2*: VEGF Receptor 2 gene
