# Supplementary figures and images for: Relaxin deficiency results in increased expression of angiogenesis- and remodelling-related genes in the uterus of early pregnant mice but does not affect endometrial angiogenesis prior to implantation
Source: Reprod Biol Endocrinol. 2016 Mar 22;14:11. doi: 10.1186/s12958-016-0148-y (PMC4802869; doi:10.1186/s12958-016-0148-y)

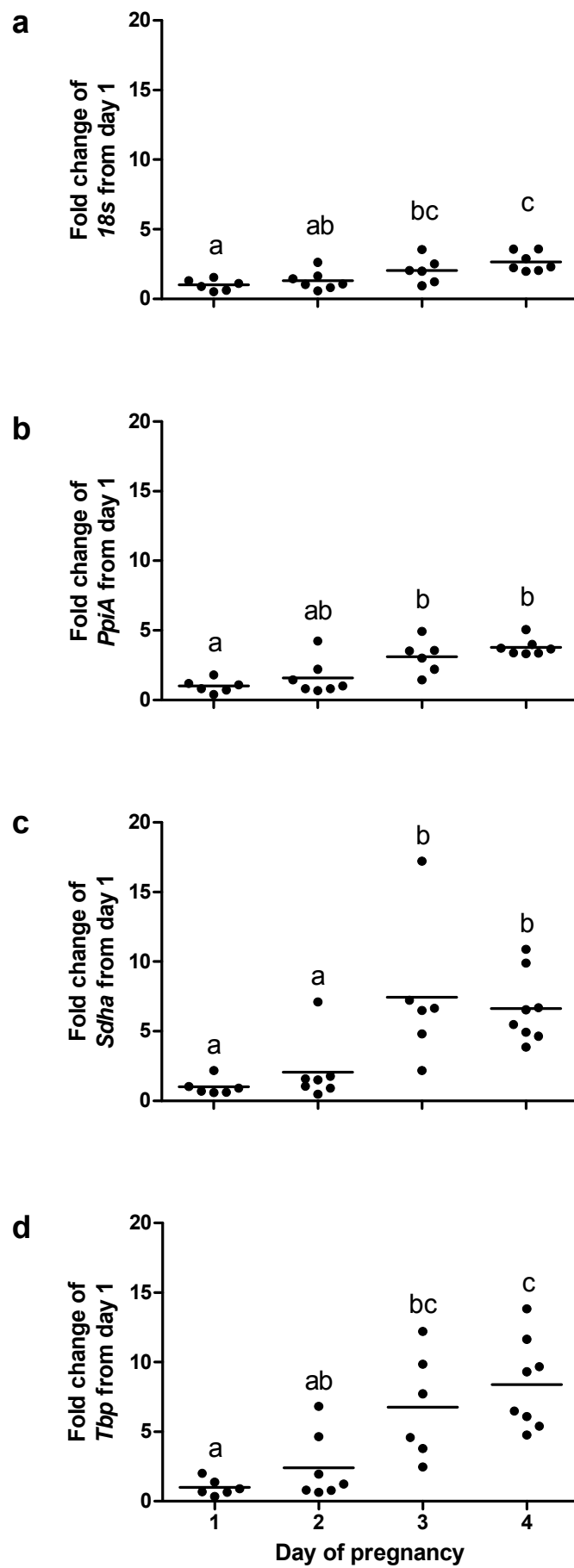

Supplement: Additional file 1: Figure S1. — Expression of (a) Rxfp1, (b) Il1β, (c) VegfA total, (d) Vegfr2, (e) Esr1, (f) Esr2, (g) Mmp14 and (h) Timp3 in the uterus of Rln +/+ and Rln -/- mice on day 6 of pregnancy (Study 3, n = 6–7). Horizontal bars indicate mean values. (PDF 35 kb) [file 12958_2016_148_MOESM1_ESM.pdf]

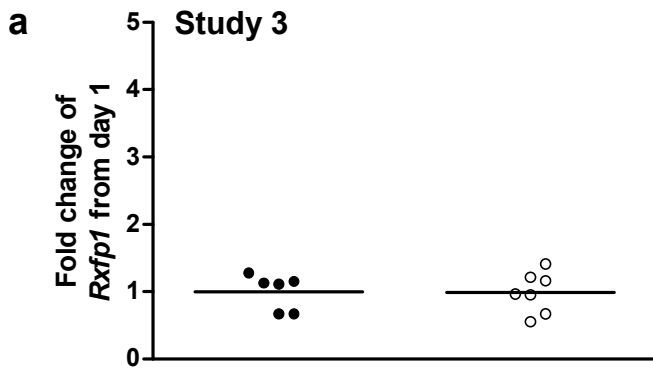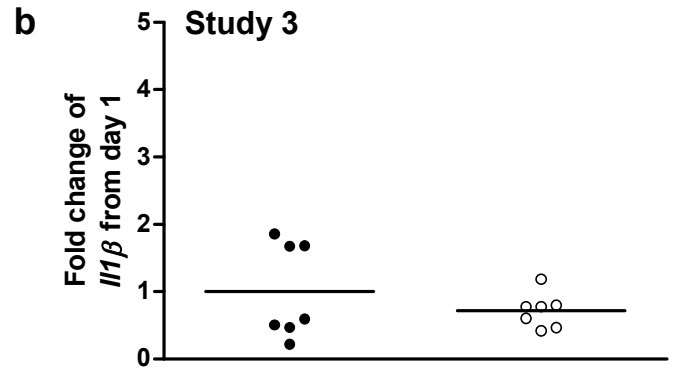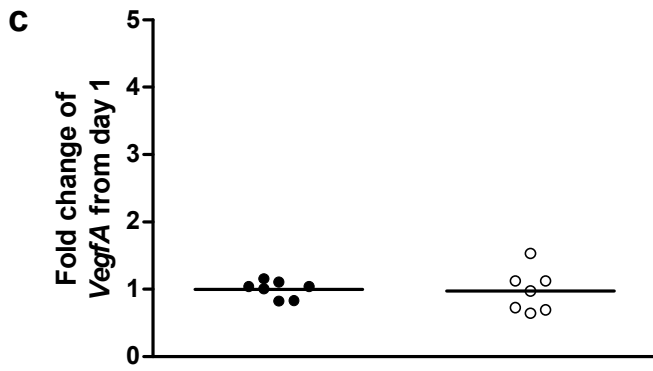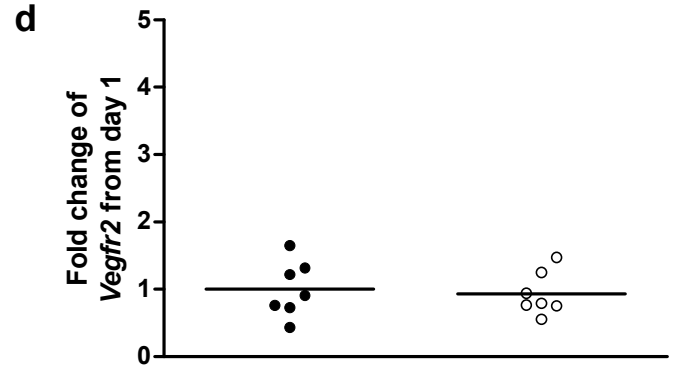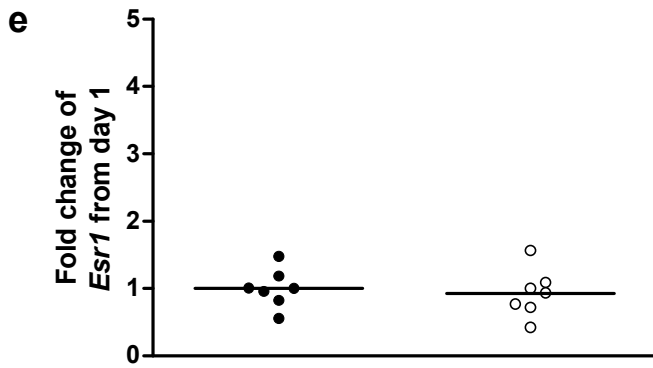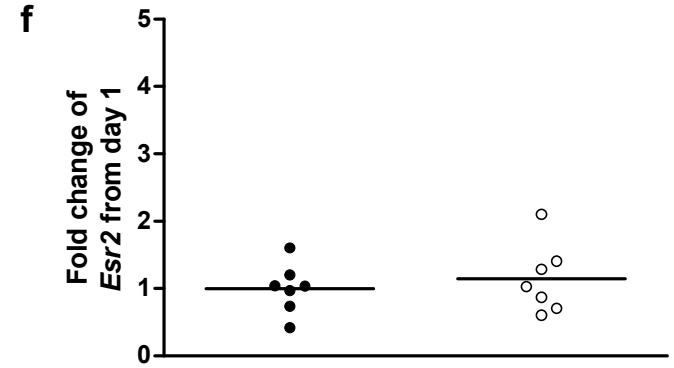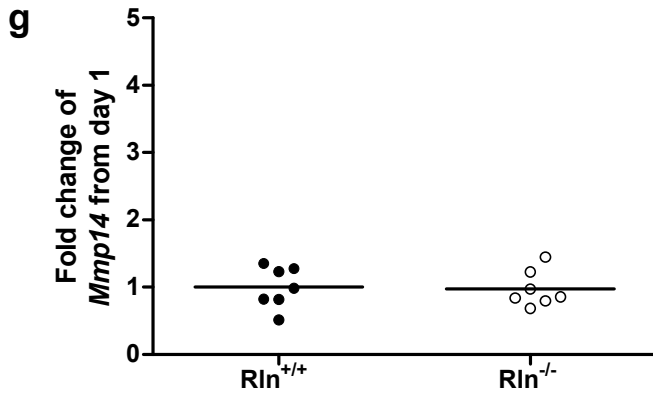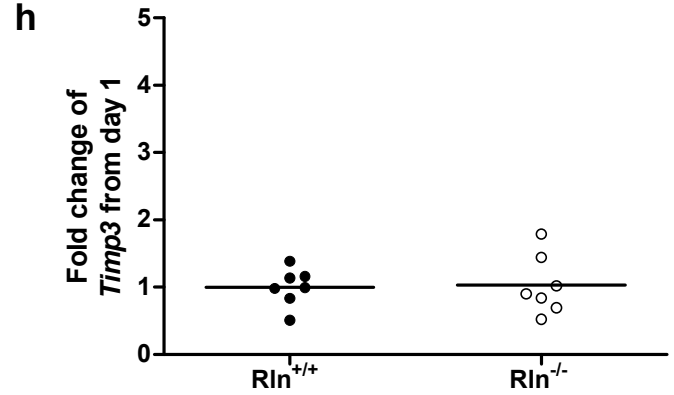

Supplement: Additional file 2: Figure S2. — Expression of (a) 18 s, (b) PpiA, (c) Sdha and (d) Tbp in the uterus of Rln +/+ mice on days 1 to 4 of pregnancy (n = 6–8). Horizontal bars indicate mean values. Groups that do not share a letter are significantly different from one another (p < 0.05). (PDF 37 kb) [file 12958_2016_148_MOESM2_ESM.pdf]
