# Supplementary material for: Relaxin deficiency results in increased expression of angiogenesis- and remodelling-related genes in the uterus of early pregnant mice but does not affect endometrial angiogenesis prior to implantation
Source: Reprod Biol Endocrinol. 2016 Mar 22;14:11. doi: 10.1186/s12958-016-0148-y (PMC4802869; doi:10.1186/s12958-016-0148-y)
Supplement: Additional file 3: Table S1. — Primer and probe sequences for the quantitative amplification of murine genes. Table S2. Mean CT values and the range of CT values for each gene analyzed by qPCR in the uterus of wildtype (Rln +/+) and relaxin deficient (Rln -/-) mice (n = 6–8). Table S3. Primer sequences for the RT-PCR amplification of murine genes. (DOC 111 kb) [file 12958_2016_148_MOESM3_ESM.doc]

**Supplementary Table 1**. Primer and probe sequences for the quantitative amplification of murine genes.

| Gene | Accession number | Oligo type | Primer sequence 5’-3’ | Amplicon length |
| --- | --- | --- | --- | --- |
| *18S* | NR_003278 | Forward  Reverse  Probe | GCATGGCCGTTCTTAGTTGG  TGCCAGAGTCTCGTTCGTTA  TGGAGCGATTTGTCTGGTTATTCCGA | 67 bp |
| *Ankrd37* | NM_001039562 | Forward  Reverse  Probe | Tggagacaggagcatcagtg  Gccttgtggagtggagtttc  acctcaaccaacaggatgtctt | 163 bp |
| *Egln1* | NM_053207 | Forward  Reverse  Probe | AGGCAACGGAACAGGCTATGTC  TGGGTTCAATGTCAGCAAACTG  gccaaggtaagtggaggtattc | 164 bp |
| *Esr1* | NM_007956 | Forward  Reverse  Probe | Ctttgatccacctgatggcc  actcatgtgccggatatggg  atcgccgcctagctcagctccttct | 82 bp |
| *Esr2* | NM_207707/  NM_010157 | Forward  Reverse  Probe | gtcagggtggttgggttgtg  atggaaagtacaacgagagccttc  tgtcccctgtaccaggacttactgctgaa | ***78 bp |
| *Hgf* | NM_010427 | Forward  Reverse  Probe | Cacctcctcctgcttcatgt  Aacgtaaagcccctgttcct  ccctatgcagaaggacagaaga | 194 bp |
| *Hif1α* | NM_010431 | Forward  Reverse  Probe | GAGGCTCACCATCAGTTATTTACG  CTGTGCCTTCATCTCATCTTCAC  AAACTTCTGGATGCCGGTGGTCTAGA | 82 bp |
| *Il1β* | NM_008361 | Forward  Reverse  Probe | TGGCAACTGTTCCTGAACTCAA  TGGAAGCAGCCCTTCATCT  TGACAGTGATGAGAATGACCTG | 106 bp |
| *Mmp14* | NM_008608 | Forward  Reverse  Probe | cttcaaggagcgatggttct  gcattgggtatccatccatc  cgggtgaggaataaccaagt | 62 bp |
| *Ppia* | NM_008907 | Forward  Reverse  Probe | Aagactgaatggctggatgg  Agctgtccacagtcggaaat  gaaggtgaaagaaggcatgaac | 140 bp |
| *Pgr* | NM_008829 | Forward  Reverse  Probe | Caatggaagggcagcataac  Cttacgacctccaaggacca  cgtctgagaaagtgttgtcagg | *134 bp |
| *Rln* | NM_011272 | Forward  Reverse  Probe | aggcaagccactgaagttgt  gtcgtatcgaaaggctctgc  gccatccttcatcaacaaaga | 62 bp |
| *Rxfp1* | NM_212452 | Forward  Reverse  Probe | gcttccactaactcctttgaggc CATGCATTGTTTGTGCCGAG  AAACTTCCGAATGCTTGGTTGGCTC | 76 bp |
| *Sdha* | NM_023281 | Forward  Reverse  Probe | ttgatgctgtggttgtaggc  ctccctgtgctgcaacagta  gccttacaaagctctttcctacc | 132 bp |
| *Tbp* | NM_013684 | Forward  Reverse  Probe | cttcgtgcaagaaatgctga  cttcactcttggctcctgtg  gccttacaaagctctttcctacc | 130 bp |
| *Timp3* | NM_011595 | Forward  Reverse  Probe | GGCCCTTTGGCACTCTGGT  GAGGCTTCCGTGTGAATGTACTG  GATGAAGATGTACCGAGGCTTC | 97 bp |
| *VegfA* | NM_001025250 | Forward  Reverse  Probe | GCACCCACGACAGAAGGA  GCTTCGCTGGTAGACATCCAT  AGCAGAAGTCCCATGAAGTGAT | 69 bp |
| *Vegfr2* | NM_010612 | Forward  Reverse  Probe | gattacttgcaggggacagc  cctgggaatggtgagtgttt  aggaaagggtattggtgactga | 133 bp |

*Designed to detect both isoforms of gene in question.

**Supplementary Table 2. Mean C_T_ values and the range of C_T_ values for each gene analyzed by qPCR in the uterus of wildtype (*Rln^+/+^*) and relaxin deficient (*Rln^-/-^*) mice (n = 6-8)**.

| Study 1 | Mean C_T_ value and range | | | |
| --- | --- | --- | --- | --- |
| Gene | *Rln^+/+^* Day 1 | *Rln^+/+^* Day 2 | *Rln^+/+^* Day 3 | *Rln^+/+^* Day 4 |
| *18s* | 14.79  (13.92-15.77) | 14.59  (13.44-15.62) | 14.42  (13.01-14.55) | 13.64  (12.99-13.84) |
| *Ankrd37* | 33.45  (30.90-34.64) | 34.88  (34.30-36.23) | 34.41  (33.57-35.11) | 33.90  (33.19-34.77) |
| *Egln1* | 30.88  (28.81-31.44) | 30.82  (29.27-32.16) | 29.82  (28.87-30.67) | 29.35  (28.4-30.26) |
| *Esr1* | 29.32  (27.67-30.21) | 28.57  (25.63-30.30) | 27.10  (26.42-28.21) | 26.65  (25.56-27.45) |
| *Esr2* | 32.01  (30.85-33.35) | 31.91  (31.85-32.78) | 32.72  (31.79-33.20) | 33.03  (32.29-33.87) |
| *Hgf* | 32.06  (28.77-34.77) | 30.36  (26.57-32.34) | 27.04  (26.44-28.32) | 26.98  (25.60-29.31) |
| *Hif1α* | 30.08  (27.32-31.1) | 29.09  (27.19-30.78) | 27.31  (26.38-28.69) | 26.91  (25.97-27.81) |
| *Mmp14* | 29.72  (26.37-31.16) | 29.02  (26.00-30.60) | 27.63  (26.91-28.7) | 27.35  (25.95-27.71) |
| *PpiA* | 28.11  (27.24-29.45) | 27.73  (26.02-28.66) | 26.57  (25.80-27.57) | 26.35  (25.76-26.37) |
| *Rln* | 37.73  (35.32-39.08) | 35.32  (34.37-36.20) | 33.06  (32.40-34.04) | 34.34  (33.69-34.24) |
| *Rxfp1* | 35.35  (33.82-36.49) | 34.20  (32.23-35.82) | 33.67  (32.72-35.21) | 33.18  (32.38-34.15) |
| *Sdha* | 29.42  (27.81-30.28) | 28.99  (26.70-30.60) | 26.90  (25.42-28.41) | 26.89  (26.08-27.58) |
| *Tbp* | 32.68  (30.65-34.31) | 32.09  (30.03-33.49) | 30.25  (29.19-31.49) | 29.82  (29.01-30.55) |
| *Timp3* | 32.48  (28.18-35.39) | 30.89  (28.17-32.53) | 28.87  (27.96-30.08) | 28.63  (27.83-28.98) |
| *VegfA* | 31.11  (29.27-31.56) | 30.96  (29.25-31.79) | 30.36  (29.40-31.44) | 29.96  (29.18-30.70) |
| *Vegfr2* | 32.34  (30.49-34.18) | 31.06  (28.95-32.17) | 29.33  (28.44-30.65) | 28.84  (28.01-29.69) |
| Study 2 | Mean C_T_ value and range | | | |
| Gene | *Rln^+/+^* Day 1 | *Rln^-/-^* Day 1 | *Rln^+/+^* Day 4 | *Rln^-/-^* Day 4 |
| *Ankrd37* | 33.28  (31.05-34.67) | 31.36  (30.61-32.30) | 33.84  (32.19-35.18) | 35.18  (33.89-36.66) |
| *Egln1* | 30.88  (28.93-31.64) | 28.61  (28.39-29.03) | 29.38  (28.75-30.23) | 29.25  (28.98-29.57) |
| *Esr1* | 29.79  (28.06-30.60) | 27.66  (27.06-29.03) | 27.10  (26.55-27.70) | 26.75  (26.38-27.08) |
| *Esr2* | 32.77  (31.54-34.48) | 33.80  (32.24-34.52) | 33.92  (32.93-34.51) | 33.82  (33.05-34.79) |
| *Hgf* | 33.02  (29.32-34.48) | 30.46  (28.15-33.72) | 27.78  (26.62-30.45) | 26.86  (26.61-27.35) |
| *Hif1α* | 29.96  (29.05-30.92) | 27.50  (26.44-29.34) | 26.68  (26.16-27.76) | 26.84  (26.58-27.09) |
| *Mmp14* | 29.38  (28.52-30.79) | 27.25  (25.71-29.06) | 27.02  (26.79-27.35) | 27.03  (26.79-27.40) |
| *Pgr* | 27.68  (25.61-30.12) | 25.64  (23.45-28.40) | 27.16  (26.22-27.99) | 24.70  (24.46-24.84) |
| *Rxfp1* | 35.81  (34.36-37.50) | 33.83  (32.45-35.49) | 33.65  (32.94-34.82) | 33.09  (32.86-33.40) |
| *Timp3* | 32.39  (28.95-35.08) | 29.88  (28.22-33.47) | 28.58  (27.67-29.27) | 27.94  (27.51-28.28) |
| *VegfA* | 30.72  (29.25-32.09) | 29.27  (28.87-29.87) | 29.51  (28.81-30.29) | 29.76  (29.41-30.04) |
| *Vegfr2* | 32.40  (30.33-34.21) | 30.56  (29.32-32.62) | 28.89  (28.25-29.85) | 28.73  (28.40-29.07) |
|  | Mean C_T_ value and range | | | |
| Gene | *Rln^+/+^* Day 6 |  | *Rln^-/-^* Day 6 |  |
| *Esr1* | 27.01  (26.39-27.81) |  | 27.1  (26.31-28.20) |  |
| *Esr2* | 33.15  (32.22-35.25) |  | 32.08  (31.08-33.00) |  |
| *Il1β* | 32.72  (31.45-35.54) |  | 32.66  (31.55-33.53) |  |
| *Mmp14* | 27.07  (26.56-27.96) |  | 26.88  (26.47-27.36) |  |
| *Rxfp1* | 28.29  (27.89-28.83) |  | 28.34  (27.75-29.15) |  |
| *Timp3* | 27.48  (26.95-28.40) |  | 27.33  (26.58-27.80) |  |
| *VegfA* | 29.08  (28.85-29.34) |  | 28.99  (28.45-29.61) |  |
| *Vegfr2* | 27.83  (26.99-28.93) |  | 27.69  (27.15-28.43) |  |

**Supplementary Table 3. Primer sequences for the RT-PCR amplification of murine genes**

| Gene | Accession number | Oligo type | Primer sequence 5’-3’ | Amplicon length |
| --- | --- | --- | --- | --- |
| *Gapdh* | NM_001289726 | Forward  Reverse | TGATGACATCAAGGTGG  TTTCTTACTCCTTGGAGGCC | 250 bp |
| *Rln* | NM_011272 | Forward  Reverse | TCTCGGAGGAGTGGATGGAC  ATCGCTCAACACAGGTGCG | 290 bp |
| *Rxfp1* | NM_212452 | Forward  Reverse | GGAGACAACAATGGGTGGTC  TCGACTGAGGTGATTGTCTTC | *344 bp  *450 bp |

*Designed to detect both isoforms of gene in question.
